# Regulation of *NR4A2* Gene Expression and Its Importance in Neurodegenerative and Psychiatric Diseases

**DOI:** 10.3390/ijms26189162

**Published:** 2025-09-19

**Authors:** Elizabeth Ruiz-Sánchez, Carolina Rojas, Petra Yescas Gómez, Nancy Martínez-Rodríguez, Ángel Alberto Ruiz-Chow, Concepción Nava-Ruiz, Gabriela Ibáñéz-Cervantes, Ivonne Maciel Arciniega-Martínez, Aldo Arturo Reséndiz-Albor, Patricia Rojas

**Affiliations:** 1 Laboratorio de Neuroquímica, Instituto Nacional de Neurología y Neurocirugía Manuel Velasco Suárez S.S., Avenida Insurgentes Sur. No. 3877, Mexico City 14269, Mexico; elizabeth.ruiz@innn.edu.mx; 2 Instituto de Investigaciones Biomédicas, Universidad Nacional Autónoma de México, Circuito Escolar s/n, Mexico City 04510, Mexico; rocastan@yahoo.com.mx; 3 Departamento de Genética, Instituto Nacional de Neurología y Neurocirugía Manuel Velasco Suárez S.S., Avenida Insurgentes Sur. No. 3877, Mexico City 14269, Mexico; petra.yescas@innn.edu.mx; 4 Unidad de Investigación Epidemiologica en Endocrinología y Nutrición, Hospital Infantil de México Federico Gómez, Dr Márquez 162, Mexico City 06720, Mexico; nmartinez@himfg.edu.mx; 5 Unidad de Neuropsiquiatría, Instituto Nacional de Neurología y Neurocirugía Manuel Velasco Suárez S.S., Avenida Insurgentes Sur. No. 3877, Mexico City 14269, Mexico; angel.ruiz@innn.edu.mx; 6 Laboratorio de Neuropatología Experimental, Instituto Nacional de Neurología y Neurocirugía Manuel Velasco Suárez S.S., Avenida Insurgentes Sur. No. 3877, Mexico City 14269, Mexico; concepcionnava@hotmail.com; 7 Departamento de Desarrollo de Tecnologías, Centro Interdisciplinario de Ciencias Marinas (CICIMAR-IPN), Instituto Politécnico Nacional, C. Instituto Politécnico Nacional s/n, La Paz 23096, Baja California Sur, Mexico; gaby_aldebaran9@yahoo.com.mx; 8 Laboratorio de Inmunonutrición, Sección de Estudios de Posgrado e Investigación, Escuela Superior de Medicina, Instituto Politécnico Nacional, Plan de San Luis y Salvador Díaz Mirón s/n, Mexico City 11340, Mexico; iarciniega@ipn.mx; 9 Laboratorio de Inmunidad de Mucosas, Sección de Estudios de Posgrado e Investigación, Escuela Superior de Medicina, Instituto Politécnico Nacional, Plan de San Luis y Salvador Díaz Mirón s/n, Mexico City 11340, Mexico; aresendiza@ipn.mx

**Keywords:** *NR4A2* gene expression, *NR4A2* epigenetic mechanisms, neurodegenerative diseases, psychiatric disorders, gene expression regulation, transcription factors, epigenetic mechanisms, neurodegenerative diseases, psychiatric disorders, central nervous system

## Abstract

Nuclear receptor subfamily 4 group A member 2 (NR4A2) is a transcription factor that regulates the expression of different genes involved in essential biological processes, including cell proliferation, neuronal development, immune response, cellular stress, apoptosis, DNA repair, and angiogenesis. The gene encoding this transcription factor is called NR4A2 and has been identified as an immediate early gene. Moreover, research in animal models and clinical trials has suggested an association between reduced *NR4A2* gene expression and some neurodegenerative diseases and psychiatric disorders. These include Parkinson’s disease, Alzheimer’s disease progression, schizophrenia, substance abuse (alcohol and amphetamines), neurodevelopmental disorders, and cognitive imairment. NR4A2 activity is controlled at multiple levels, including transcriptional and post-transcriptional regulation of its gene expression, such as translational and post-translational processes. This review summarizes the current knowledge of the *NR4A2* gene, encompassing its structure and the molecular mechanisms that regulate its expression. The key epigenetic mechanisms that regulate its gene expression are emphasized, including DNA methylation, histone deacetylation, and regulation by microRNAs. It also addresses its role in central nervous system pathologies associated with dysregulation of *NR4A2* gene expression. Finally, we discuss the potential of these regulatory mechanisms as biomarkers and therapeutic targets for neurodegenerative diseases and psychiatric disorders.

## 1. Introduction

NR4A2 (also known as NURR1) belongs to the NR4A subfamily of nuclear receptors, along with NR4A1 (NUR77) and NR4A3 (NOR-1). These transcription factors (TFs) are activated in response to a wide range of stimuli and stressors. They are expressed in a variety of tissues and play important roles in regulating numerous and diverse physiological and pathological processes, including metabolism, cell cycle, carcinogenesis, inflammation, vascular processes, immune response and neuronal function [1,2,3,4]. These nuclear receptors are regarded as “orphans” due to the occupation of the ligand-binding site (LBD) by bulky and compact hydrophobic residues, limiting the capacity of ligands to binding. Nevertheless, some endogenous and exogenous molecules have been identified that can modulate the activity of these TFs [5,6].

The mechanisms regulating the transcriptional activity of this family of nuclear receptors are not yet fully understood, but they have been shown to involve post-translational processes and regulation of their gene expression [1,7]. In the context of neurodegenerative diseases and psychiatric disorders, it is therefore important to highlight the role of the *NR4A2* gene and its gene regulation.

NR4A2 plays an important role in various biological processes, such as cell proliferation, neuronal development, immune response, cellular stress response, apoptosis, DNA repair, and angiogenesis [8,9,10]. In the nervous system, NR4A2 participates in neuroprotection and plays a critical role in the differentiation, maintenance and function of dopaminergic (DAergic) neurons [11]. Dopamine is an important neurotransmitter involved in the modulation of motor and cognitive functions, as well as motivation and reward [12]. NR4A2 also participates in hippocampal synaptic plasticity [13], which is crucial for diverse cognitive functions, particularly learning and memory. In particular, NR4A2 has been implicated in Alzheimer’s disease (AD) progression, Parkinson disease, schizophrenia, substance abuse (alcohol and cocaine), neurodevelopmental disorders and cognitive impairment among others [7,13,14,15,16,17,18]. This TF is also involved in anti-inflammatory, and homeostatic processes in neurons and non-neuronal cell [19].

Given its importance, understanding the mechanisms that regulate *NR4A2* gene expression and identifying potential therapeutic targets for neurological diseases and psychiatric disorders is a priority.

This review is therefore organized as follows: The first section provides an overview of the NR4A2 TF characteristics. The second section analyzes *NR4A2* gene expression across different tissues to provide a comprehensive overview of its expression patterns. Subsequently, we analyze the regulation of *NR4A2* gene expression, including its gene structure, and the cis (promoter) and trans regulatory factors involved in its gene expression. This section also highlights the epigenetic and post-transcriptional mechanisms that regulate *NR4A2* gene expression. These mechanisms are key for understanding the complex process of gene expression. Finally, we describe the dysregulation of *NR4A2* gene expression in various neurodegenerative and psychiatric diseases. This allows us to discuss NR4A2 as a potential therapeutic target and the challenges of potential treatment involving NR4A2.

## 2. Methods

This review was conducted using electronic databases including PubMed, Scopus, and Google Scholar, focusing on peer-reviewed literature published in English between 2020 and 2025. Furthermore, relevant references were selected based on their contribution to the understanding of NR4A2-related mechanisms in neurobiology and disease. Although the initial search criteria were limited to recent publications, several of these sources cited foundational studies from earlier decades, which were subsequently incorporated for the importance of supporting mechanistic interpretations. The search strategy included the used of specific key terms such as NR4A2 gene expression; NR4A2 and expression regulation; NR4A2 and epigenetic mechanisms; *NR4A2* and DNA methylation; *NR4A2* and histone modification; *NR4A2* and genetic variants; NR4A2 and neurodegenerative diseases; NR4A2 and psychiatric disorders; NR4A2 and Parkinson’s disease; NR4A2 and Alzheimer’s disease; NR4A2 and neurodevelopmental disorders; NR4A2 and schizophrenia; NR4A2 and major depressive disorder; NR4A2 and substance use disorders.

## 3. NR4A2 Protein

TFs are proteins that regulate the process of transcription through the binding of specific DNA sequences, known as response elements, located throughout the genome. Once bound to DNA, they allow the direct recruitment of TFs to the pre-initiation complex or the binding of intermediate factors, also called co-regulator proteins which are positive modulators (co-activators) and negative modulators (co-repressors) in order to activate or repress the expression of target genes [20]. These co-regulators can form subunits of multiprotein complexes that act at different functional levels, such as chromatin remodeling, histone modification or preinitiation complex modulation. Consequently, co-regulators regulate the transcription of their target genes [21].

Nuclear receptors interact with co-regulatory proteins through activation domains. Many nuclear receptors have at least two activation domains: a ligand-independent activation domain (AF-1), located in the N-terminal region, and a ligand-dependent activation domain (AF-2), in the C-terminal region [22].

**Figure 1:**
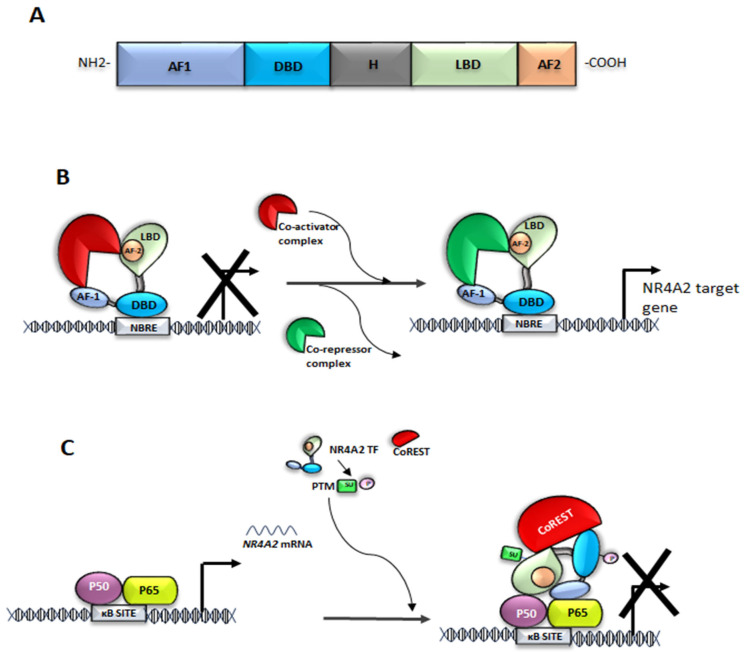
Structure and function of NR4A2 TF. (**A**) Schematic representation of the NR4A2 functional domains: N-terminal region AF-1, a conserved DBD, a H region and a C-terminal LBD-AF2 domain [23]. NR4A2 TF regulates gene expression through two main processes. (**B**) NR4A2 TF binds to specific DNA sequences through its DBD, thereby regulating transcription. Once bound to DNA, NR4A2 can bind to a co-regulator to regulate gene expression [24,26]. (**C**) NR4A2 binds to other TFs or co-regulators, without directly interacting with DNA. NR4A2 interacts with the p65 subunit of NF-κB in a manner determined by its post-translational modifications, such as sumoylation (mediated by STAT4) and phosphorylation (mediated by a Nemo-like kinase). NR4A2 then binds to the phosphorylated p65 subunit of NF-κB (mediated by GSK-3) in the promoter regions of target genes. This interaction leads to the recruitment of a repressor complex and affects the transcription of NF-κB target genes [27]. TF, transcriptor factor; DBD, DNA-binding domain; H, hinge region: LBD, ligand-binding domain; AF1, activation function 1; AF2: activation function 2; SU, sumoylation; P, phosphorylated; κB SITE, response element for NF-κB; (p50, p65), NF-κB, nuclear factor kappa B; NBRE, NGFI-B response element; CoREST repressor complex, co-repressor for element-1-silencing transcription factor.

As a member of the NR4A family, NR4A2 shares structural homology with nuclear receptor domains (Figure 1A). These include: (a) a modulatory domain called the activation function AF-1 N-terminal domain; (b) a conserved double zinc finger DNA-binding domain (DBD); (c) a flexible hinge region; (d) a ligand-binding domain (LBD) composed of 12 helices; and (e) the AF-2 C-terminus, which interacts with a co-regulatory protein [23]. NR4A2 lacks a ligand binding pocket and a canonical nuclear receptor binding site for co-regulatory proteins, leading to changes in gene expression [24]. The available studies suggest that the LBD of NR4A2 may function in an atypical ligand-independent manner, behaving dynamically and likely capable of expanding to bind ligands. Furthermore, some NR4A2 ligands function independently of binding to its LBD, and it has been proposed that NR4A2 transcriptional activity is modulated by ligands that target its heterodimer binding partner, such as retinoid X receptor alpha [25].

The NR4A2 TF binds DNA sequences functioning as monomers (Figure 1B), homodimers or heterodimers, enabling regulation of multiple genes. The structure of the NR4A2 TF permits the regulation of gene expression of several target genes, which are essential for the development, maintenance, and survival of DAergic neurons [26]. Additionally, this nuclear receptor is also involved in maintaining cellular homeostasis, mitochondrial function and regulating the anti-inflammatory response in adult cells [2,28,29]. Therefore, this important TF is essential for different functions, which underlines the need for effective regulation of its activity across diverse mechanisms in tissues and cells.

The mechanisms that regulate NR4A2 activity include its genetic expression, post-transcriptional, translational, and post-translational processes such as ubiquitination, phosphorylation, and SUMOylation. The last three mechanisms mentioned have been revised by other authors [30,31]. In addition, *NR4A2* expression and localization have been described in both the cytoplasm and nucleus, changes in their cellular localization or heterodimerization with specific proteins would also make it possible to control their stability and thus their activity [31]. In this review we describe the *NR4A2* gene and the molecular mechanisms that regulate the transcription and post-transcriptional processes of the gene encoding NR4A2 TF.

## 4. *NR4A2* Gene Expression

NR4A2 TF is encoded by an immediate early gene (IEG), called *NR4A2*. This gene is also known as *NURR1*, *NOT*, *HZF-3*, *TINUR*, and *RNR1*. It is important to note that the expression of IEGs occurs rapidly (in minutes) and is transient. Furthermore, IEGs respond to a variety of extrinsic and intrinsic stimuli in diverse cell types, suggesting a general response mechanism [32,33]. Many IEGs encode TFs and DNA-binding proteins that regulate the expression of target genes, which are referred to as late response genes [34]. In the central nervous system (CNS), IEG expression plays an important role in various processes, including brain development, learning, neuronal plasticity, memory formation, and response to drugs abuse [33,35]. The *NR4A2* IEG is particularly involved in all these processes.

Therefore, in response to cellular stimulus, the *NR4A2* gene is expressed ubiquitously with rapid transcription. However, dysregulated expression of this IEG has been associated with the understanding of molecular mechanisms and therapeutic targets in neurological, psychiatric, and metabolic disorders [36,37].

Notably, gene expression mapping in 40 different normal human tissues identified *NR4A2* messenger ribonucleic acid (mRNA) expression (GTEX project; HPA project) [38].

Accordingly, *NR4A2* mRNA expression has been documented in a range of tissues across diverse species. For example, the Human Protein Atlas version 23 [38] has reported *NR4A2* gene expression in mammals, including human, pig and mouse. In particular, *NR4A2* mRNA expression in human tissues, such as the adrenal gland, ovary and bone marrow, is considered enhanced by at least four-fold relative to the average expression levels observed in other tissues, regions, and cell types. Additionally, its expression has been observed in human tissues associated with metabolic functions, including the pancreas and liver, as well as in cardiac muscle tissue. However, mRNA expression of this gene in the human, pig, and mouse brain is considered to be of low regional specificity (normalized protein-transcripts per million = nTPM ≥ 1 in at least one tissue/region/cell type but not elevated in any tissue/region/cell type) [38,39,40].

**Figure 2:**
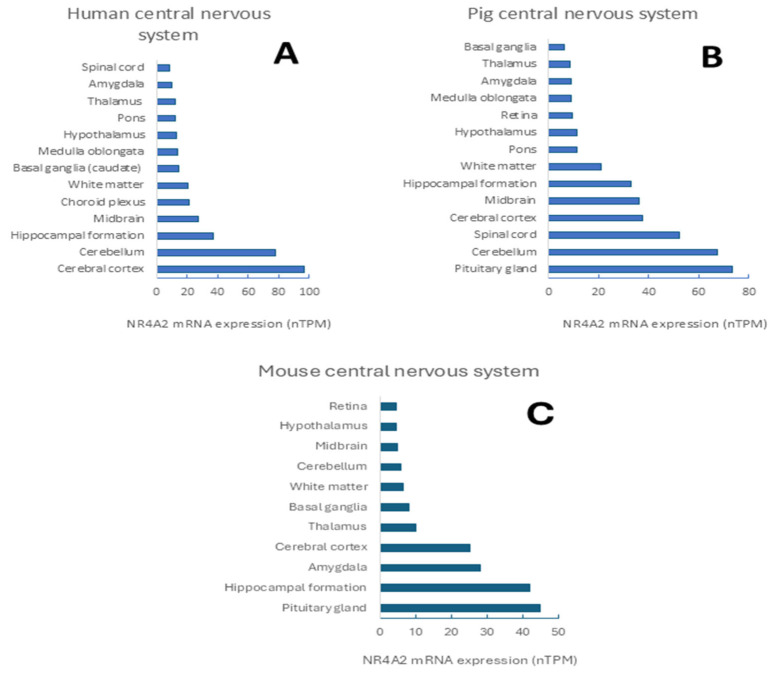
Regional variation in *NR4A2* mRNA expression in the central nervous system of human (**A**), pig (**B**) and mouse (**C**). The reported data were obtained from the Human Protein Atlas (HPA) based on an RNA-sequencing (RNA-seq) dataset for the brains of human, pig and mouse. Data are based on normalized expression (nTPM) values according to HPA RNA-seq brain gene data, which are available on the open-access resource http://v20.proteinatlas.org/humanproteome/brain (accessed on 7 August 2025) [38]. nTPM = normalized protein-coding transcripts per million.

Notably, the distribution of *NR4A2* mRNA expression in different brain regions of three mammalian species (human, pig, and mouse) differed when subregions were analyzed (Figure 2). For example, *NR4A2* mRNA expression in the cerebral cortex is the highest in the human brain and its protein expression is high in neuronal cells and glia, as well as medium in endothelial cells in that brain region. The second brain region with the highest expression of *NR4A2* mRNA is the cerebellum, which also exhibits a high expression of the protein, particularly in the cells of the molecular layer. Also, the protein is expressed at a medium level in Purkinje and granular layer cells. Thus, the mRNA expression of human CNS shows the following order: cerebral cortex > cerebellum > hippocampal formation > midbrain > choroid plexus > white matter > basal ganglia > medulla oblongata > hypothalamus > pons > thalamus > amygdala > spinal cord [38,39].

The mRNA expression pattern of *NR4A2* mRNA for the pig CNS is as follows: pituitary gland > cerebellum > spinal cord > cerebral cortex > midbrain > hippocampal formation > white matter > pons > hypothalamus > retina > medulla oblongata > amygdala > thalamus > basal ganglia. In the case of the mouse CNS, the following pattern is observed: pituitary gland > hippocampal formation >amygdala > cerebral cortex > thalamus > basal ganglia > white matter > cerebellum > midbrain > hypothalamus > retina [38].

## 5. Expression Regulation *NR4A2*

Transcription is the process by which information from a specific DNA segment is copied into an RNA molecule. This represents the initial step of gene expression and plays a crucial role in determining both timing and the mechanism by which functional products, such as proteins and some RNAs, are produced [41].

Transcription involved a complex cascade of events, orchestrated by a multitude of molecular players that form interconnected regulatory networks. These events range from specific DNA-protein interactions to the recruitment and assembly of nucleoprotein complex. Since most signaling pathways converge on the transcriptional machinery, transcriptional dysregulation has been implicated in a variety of human diseases [42].

The regulation of transcription is multifactorial, involving cis-regulatory elements (e.g., promotor region) and trans-acting factors (e.g., transcription factors, co-regulators and chromatin-modifying enzymes). Additionally, epigenetics mechanisms -such as DNA methylation, histone modification, and non-coding RNA including MicroRNA (miRNAs)-, play essential role in modulating gene expression without altering the underlying DNA sequence [43].

### 5.1. NR4A2 Gene and Cis Regulatory Elements (Promoter)

#### 5.1.1. *NR4A2* Gene

**Figure 3:**
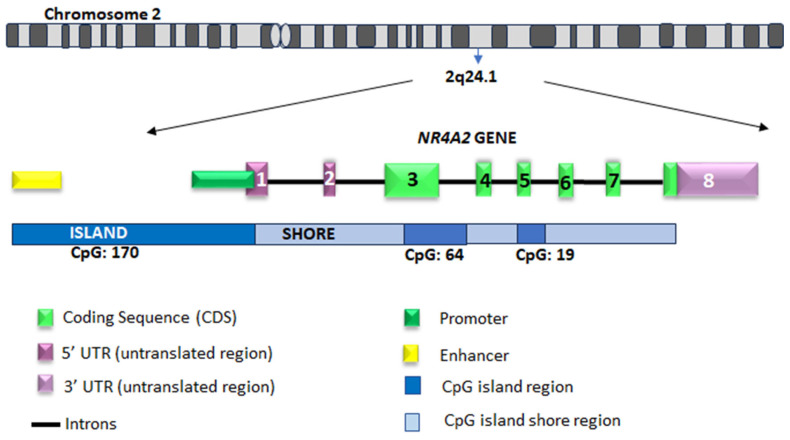
*NR4A2* gene representation. Gene is located on chromosome 2, band q24.1. It consists of 8 exons and 7 introns. The *NR4A2* gene contains different CpG islands that extend through its regulatory regions (promoter and enhancer), as well as in the body of the gene [50,51]. The numbers represent exons 1 to 8.

In humans, this TF is encoded by the *NR4A2* gene (also known as *NURR1*), located on chromosome 2q24.1 [44,45]. This gene exhibits high homologous with its murine and porcine orthologs [46,47]. Structurally, *NR4A2* consists of a promoter region, eight exons, and seven introns (Figure 3). The open reading frame spans from exon 3 to the 5′-end of exon 8, encompassing 1794 base pairs, and encodes a 598 amino acid with a molecular weight of approximately 66 kDa [45,48]. The translation initiation site is located at the start of exon 3, and the stop codon is situated at the 5′ end of exon 8. The 5′-untranslated region (UTR) encompasses the initial two exons. The 3′UTR at the 3′ end of exon 8 contains ATTA repeats [47]. These UTR sequences are essential for stabilizing mRNA and giving it a short half-life, a characteristic of IEG [49].

#### 5.1.2. Promoter Region

The *NR4A2* promoter region exhibits a high degree of sequence conservation among humans, mice, and pigs [47]. It is located upstream of the transcription start site and characterized by a high concentration of CpG dinucleotides [46]. Specifically, the *NR4A2* promoter contains five CpG islands, while the first exon contains one additional island. Furthermore, the gene body harbors a differential methylation region (DMR) between exon 2 to 5, where two CpG islands overlap [47,52,53].

Among the transcriptional regulatory elements of the *NR4A2* promoter, the cAMP response elements (CREs) are particularly relevant, as they serve as binding sites for the CRE binding protein (CREB). These sequence in the *NR4A2* promoter region are critical for memory and learning processes, particularly in hippocampus through the CREB signaling pathway [13]. In addition, these sequences have been associated with the expression of neuroprotective genes, such as *NR4A2* gene, which play key role in preserving neuronal integrity following stress or injury [54].

In the mouse, six CRE sites have been identified in the *NR4A2* promoter region. Three of these are distal CRE half sites (sequences located between −3 Kpb to −500 bp from the transcription start site, TGACG/CGTCA). In the proximal position (−500 bp to 300 bp), there is one full CRE site conserved between species (TGACGTCA/TGACGTCA) and two half CRE sites. These three proximal CRE sites have a TATA box within 300 bp and together are called the CRE/TATA promoter region. These regulatory sequences are important in activity-dependent gene regulation, particularly in response to neuronal activity and cAMP signaling. A notable example is the involvement of these sequences in *NR4A2* gene expression in neurons. The presence of these elements allows preferential transcription of the *NR4A2* gene when neurons are stimulated by depolarization and cAMP [55].

In the human *NR4A2* promoter region, consensus sequences for the binding of both Nuclear Factor Kappa B (NF-κB) (−585/−576) and CREB (−171/−163) have been identified. Furthermore, CREB and NF-κB consensus sites exhibit high conservation within the *NR4A2* promoter region of both human and mouse. This finding suggests that these binding sites play a critical functional role. This is supported by their very high evolutionary conservation [56].

Additionally, the promoter also contains response elements for transcription factors such as GATA1, SRY, MZFI, Sox5 and CArG-like [47].

#### 5.1.3. *NR4A2* Genetic Variants

A genetic variant is a change in the DNA sequence. There are different types of genetics variants, such as single nucleotide variants (SNVs), and structural changes [57,58]. SNV are the most common variants and have been identified as risk factor for disease [58,59]. The biological effects of variants genetics depend on whether they are located in coding or non-coding regions. This may influence changes in the structure of the encoded proteins, post-transcriptional modifications, or changes in gene expression [59,60]. In addition, the context of these genetic variants is critical for identifying their effects or predicting their risk [61]. Therefore, the effect of a variant also depends on genetic interactions (epistasis), tissue and developmental stage-specific effects, as well as environmental influences [61,62]. There is increasing evidence that environmental components trigger responses that are modulated by genetic variants, especially during embryonic development [63].

In this respect, pathogenic variants in the *NR4A2* gene have been identified as a risk factor for alterations in the development of the nervous system [64]. These variants are associated with mechanisms of *NR4A2* haploinsufficiency, leading to a reduction in NR4A2 expression [65]. Similarly, genetic variants associated with familial Parkinson’s disease have been identified to reduce *NR4A2* gene expression [66].

Furthermore, *NR4A2* genetic variants have been implicated as a risk factor for several neurological and psychiatric disorders, including Parkinson’s disease, depression, schizophrenia and substance abuse [67,68,69,70].

### 5.2. Transcription Factors Regulating NR4A2 Gene Expression

**Figure 4:**
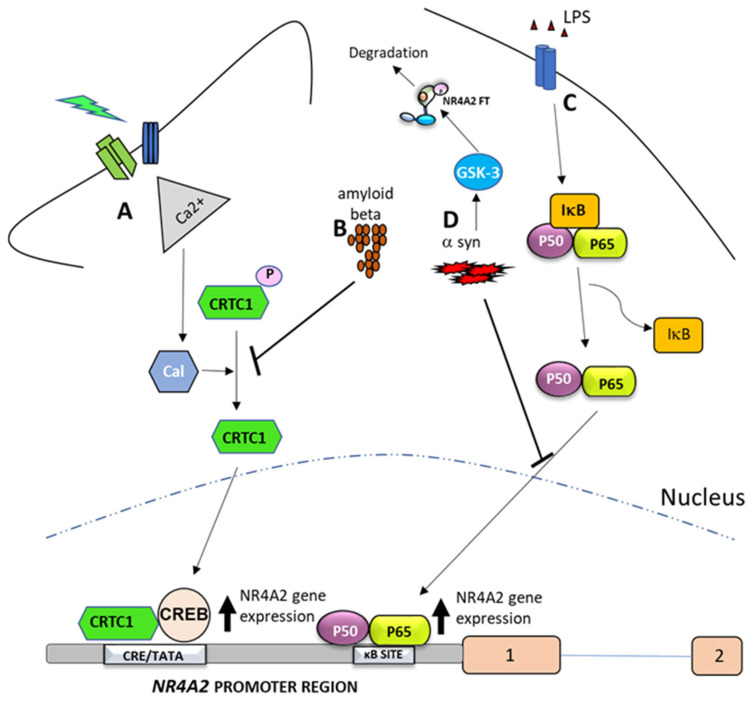
CREB and NFκB are transcription factors that regulate NR4A2 gene expression. (A) Activity-dependent induction of *NR4A2* gene expression in hippocampal neurons depends on Ca^2+^ influx through iGluRs and requires the CREB-CRTC1 signaling pathway. Neuronal activity induces CRTC1/CREB-dependent transcription through a mechanism involving the CRTC1 dephosphorylation, nuclear translocation, and CREB binding, preferentially into CRE/TATA-containing promoters [14,72]. (B) The CRTC1-dependent regulation of *NR4A2* gene expression is suppressed by the presence of amyloid beta. This mechanism is mediated by a reduction in calcium influx and disruption of CRTC1 dephosphorylation [72,73]. (C) TLR4 activation using LPS promotes NF-κB activation in nuclear localization, followed by the induction of *NR4A2* gene expression [56,74]. (D) α-synuclein inhibits NF-κB activity through the NF-κB related nuclear pathway. This reduces the binding of NF-κB to the *NR4A2* promoter, thereby affecting *NR4A2* gene expression. Moreover, GSK3 is promoted to degrade the NR4A2 protein by α-synuclein [75,76,77]. CREB, cyclic AMP response element-binding protein; (p50, p65), NF-κB, nuclear factor kappa B; α-syn, alpha synuclein; numbers 1 and 2, exons; Cal, calcineurin; LPS, lipopolysaccharides; IκB, inhibitory protein. CRTC1, CREB-regulated transcription co-activators 1; GSK3, Glycogen synthase kinase 3; CRE/TATA, sequences in the regulatory region that contain both CRE and TATA response elements.

CREB and NF-κB are among the most important TFs regulating *NR4A2* gene expression (Figure 4), highlighting the importance of these TFs in orchestrating cellular responses to different stimuli. The binding of CREB and NF-κB to the *NR4A2* promoter region is not constant, but both TFs regulate *NR4A2* expression in a cell-, tissue- and time-specific manner [27,71].

CREB is a TF that is activated by various stimuli, including cAMP and other signaling pathways or stimuli such as neuronal activity. The binding of CREB to the *NR4A2* promoter region activates its expression, particularly in areas involved in synaptic plasticity and memory, such as the hippocampus. This process is crucial for memory and learning mechanisms (Figure 4A) [13,72]. Furthermore, in the context of *NR4A2*, CREB has been identified as a regulatory factor for *NR4A2* gene expression in response to stimuli such as stress and hypoxia [54].

CREB regulated transcription co-activator 1 (CRTC1), is a transcriptional co-activator that enhances CREB-mediated transcription by interacting with the bZIP domain of CREB. It activates transcription through both consensus and variant CRE sites and increases CREB binding to RNA polymerase II [78]. Neuronal activity has been shown to induce CRTC1/CREB-dependent transcription through a mechanism involving the co-activator CRTC1, its dephosphorylation, nuclear translocation and binding to CREB preferentially into proximal CRE/TATA-containing promoters [55,72,73]. Thus, depolarization and cAMP signals induce the preferential transcription of activity-dependent genes containing promoters with proximal CRE/TATA sequences, such as *NR4A2* [55]. The binding of CRTC1 to CREB/CBP in the proximal CRE/TATA-containing *NR4A2* promoter region is an important step for robust activity-dependent *NR4A2* gene transcription in neurons. This mechanism has been involved in NR4A2-mediated glutamatergic synaptic plasticity in the hippocampus (Figure 4A) [14,72].

Notably, the CRTC1-dependent regulation of *NR4A2* gene expression is suppressed by the presence of amyloid beta in an animal model of Alzheimer’s disease [73]. This mechanism is mediated by a reduction in calcium influx and disruption of CRTC1 dephosphorylation, a process that is required for the activation of *NR4A2* gene expression (Figure 4B) [72]. These findings suggest that CRTC1 plays a key role in coupling synaptic activity to gene transcription required for hippocampal-dependent memory, and that beta-amyloid may alter cognition by affecting CRTC1 function on *NR4A2* expression [73].

On the other hand, NF-κB and NR4A2 are involved in the regulation of inflammation and immune responses [2,79]. Several studies suggest that NR4A2 and NF-κB are involved in a feedback mechanism [27,56,74,80,81,82,83,84,85,86], in which NF-κB regulates *NR4A2* expression by binding to the *NR4A2* promoter (Figure 4C) [56,74]. Conversely, NR4A2 FT modulates NF-κB activity through direct binding to response elements at the promoters of NF-κB target genes or through co-regulators of NF-κB activity (Figure 1C) [82,83,84,85]. Depending on the specific context and the association of NR4A2 with co-repressors or co-activators, this can lead to either increased or decreased expression of NF-κB target genes such as *NR4A2* [86]. Another mechanism by which NR4A2 regulates NF-κB is through its interaction with NF-kB/p65 and inhibition of its nuclear translocation [27]. Consequently, NR4A2 plays a critical role in the TLR4-NF-κB signaling pathway, which is activated by α-synuclein in BV-2 cells, a type of glial cell [87].

On the other hand, α-synuclein is an abundant pre-synaptic protein that can misfold and polymerize to form toxic fibrils that coalesce to form pathological inclusions such as Lewy bodies and Lewy neurites [88]. These pathological inclusions are present in neurodegenerative diseases such as Parkinson’s disease. In this context, the relationship between NR4A2 and α-synuclein has been reported [29,89]. During normal human aging, *NR4A2* and α-synuclein follow an inverse expression relationship in dopaminergic neurons. Thus, α-synuclein expression increases in the substantia nigra [90], while *NR4A2* expression levels progressively decrease with age [91]. Furthermore, it has been reported that α-synuclein inhibits *NR4A2* expression through mechanisms involving the NFκB TF (Figure 4D) [75]. Specifically, studies show that overexpression of α-synuclein can attenuate the activity or reduce the protein levels of NF-κB and reduce the binding of NF-κB to the *NR4A2* promoter [75,76].

In addition, glycogen synthase kinase 3 (GSK-3), which can phosphorylate NR4A2, is also activated by α-synuclein. This post-translational modification can lead to the degradation of FT NR4A2 via the ubiquitin-proteasome pathway [77]. Additionally, GSK3β regulates NF-κB signaling. Specifically, it modulates the activity of IKKγ/NEMO, a critical component of the NF-κB activation complex. GSK3β phosphorylates NEMO, affecting its stability and interactions with other proteins involved in NF-κB activation, ultimately affecting the overall activity of the signaling pathway [92]. These data suggest that α-synuclein may disrupt the NF-κB pathway, leading to reduced *NR4A2* gene expression, such as degradation of FT NR4A2 by GSK-3 (Figure 4D).

A potential feedback loop has been identified between the NR4A2 and α-synuclein. This loop suggests a reciprocal relationship in which α-synuclein can affect *NR4A2* expression. Conversely, NR4A2 activity has been shown to affect α-synuclein levels [93]. Activation of the NR4A2 TF by an agonist has been shown to attenuate altered α-synuclein gene expression [94]. Taken together, these data show that NR4A2 has a dual role in regulating the inflammatory response and α-synuclein levels. This establishes NR4A2 as a critical neuroprotective factor whose dysfunction contributes to the progression of Parkinson’s disease (PD) [95].

**Figure 5:**
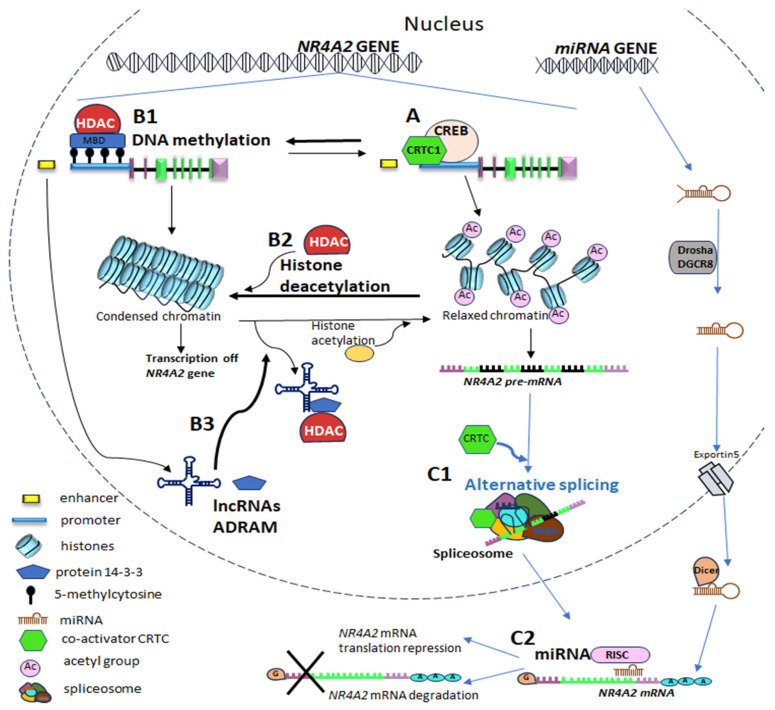
The *NR4A2* gene expression is regulated by transcriptional, epigenetic, and post-transcriptional mechanisms. (A) Transcriptional mechanisms. Regulatory sequences, such as promoters and enhancers, as well as transcription factors and co-regulators, control transcription of the *NR4A2* gene. These bind to specific sequences within the regulatory regions of the *NR4A2* gene. (B) Epigenetic mechanisms are a form of gene expression regulation that occurs without any change to the DNA sequence. (B1) DNA methylation in specific regions, such as the promoter, facilitates the binding of DNA to proteins like MBD (methyl-CpG binding domain) and HDAC. This results in the condensation of chromosomes, leading to a reduction in gene expression. In contrast, DNA methylation in the gene body can increase expression or influence alternative splicing. (B2) HDAC3, a histone deacetylase, reduces *NR4A2* expression by deacetylating histones in the *NR4A2* promoter region [96,97,98,99,100,101,102]. (B3) Non-coding RNAs, such as *ADRAM* LncRNA, can regulate enzymes or proteins that modify chromatin condensation. *ADRAM* acts in conjunction with a chaperone to move HDAC3 from the promoter region of *NR4A2* [103]. (C) Post-transcriptional mechanisms are implicated at the level of the mRNA molecule. (C1) The co-activator CRTC1 is involved in the alternative splicing of *NR4A2* mRNA [104]. (C2) Several microRNAs (miRNA) have been identified that affect in *NR4A2* expression by either inhibiting translation or contributing to *NR4A2* mRNA degradation [105,106,107,108,109]. Various proteins and protein complexes are involved in microRNA regulation, including Drosha, DGCR8, Exportin5, Dicer, and RISC. MBD, methyl-CpG binding domain, HDCA 3, Histone deacetylase 3; CRTC, CREB-regulated transcription co-activators 1; LncRNA, long non-coding RNA; ADRAM, activity-dependent lncRNA associated with memory; CREB, cyclic AMP response element-binding protein.

It is important to note that the initiation of *NR4A2* gene transcription occurs with TFs and co-regulators, described above, bind to specific sequences within the *NR4A2* gene regulatory regions such as promoters and enhancers to control NR4A2 gene expression (Figure 5A).

### 5.3. Epigenetic Mechanisms in NRA42 Gene Expression Regulation

#### 5.3.1. DNA Methylation

The process of DNA methylation consists of the covalent addition of a methyl group at the 5′ positions of DNA cytosine, resulting in the formation of 5-methylcytosine (5mC).

This mechanism occurs mainly in genomic regions rich in CpG dinucleotides, commonly referred to as CpG islands. These regions are found primarily within gene promoters in mammals, where methylation is associated with transcriptional inactivation (Figure 5(B1)) [110]. Nevertheless, DNA methylation in gene bodies is generally associated with gene expression and can influence transcription, splicing and other aspects of gene expression. The process of DNA methylation of exons plays a regulatory role in the alternative cleavage and polyadenylation of mRNAs, and this is facilitated by a protein known as CCCTC-binding factor (CTCF), which is a methylation-sensitive insulator protein. Of the various epigenetic modifications, 5mC has been the subject of the most extensive research.

In this particular context, the DMR of *NR4A2* gene has shown specific differences based on the brain region. DMR is more methylated in the hippocampus (71.6%) than in the hypothalamus (39.9%) [53]. The hippocampus is a brain region that requires constant modifications in gene expression and robust alternative splicing in order to satisfy its synaptic activity, especially in cognition [111]. These findings suggest that methylation plays a significant role in regulating *NR4A2* alternative splicing and that gene expression *NR4A2* may be influenced by region-specific epigenetic modifications. This regulation occurs through multiple mechanisms, including the direct influence of methylation on splicing factor binding and the indirect influence through altering chromatin structure, which in turn affects splicing [112].

Accordingly, it has been reported in animal models, that exposure to dieldrin, a pesticide, during development can result in differential sex-specific body methylation of the *NR4A2* gene. Specifically, dieldrin-induced *NR4A2* hypomethylation was observed exclusively in female mice and was accompanied by increased *NR4A2* expression [113].

In human studies, the methylation profiles in peripheral blood leukocytes were compared between experienced meditators with over 10 years of experience and individuals with no meditation experience [52]. The study showed a reduction in *NR4A2* gene methylation levels in experienced meditators compared to controls [52].

This evidence supports the hypothesis that methylation can influence the tissue- and time-dependent mechanisms of NR4A2 transcription and splicing.

#### 5.3.2. *NR4A2* Genetic Expression Through Histone Modification

Post-translational modifications of histones affect gene transcription and cause changes in chromatin structure. One of the most important and widely studied mechanisms of histone modification is histone acetylation. This process involves the addition of an acetyl group to a lysine residue at the amino terminus of a histone. It primarily affects chromatin relaxation [114]. The addition of an acetyl group neutralizes the positive charge of lysine, reducing the binding between histones and DNA. This results in a more open and accessible structure for the transcriptional machinery, which in turns leads to transcriptional activation. In contrast, histone deacetylases (HDAC) are a class of enzymes that remove acetyl groups from lysine residues on histones, thereby reducing gene expression (Figure 5(B2)) [115].

The regulation of transcription is indeed crucial for long-term memory formation, but not for short-term memory. Short-term memory can be established by local synaptic changes, like phosphorylation or addition of receptors, while long-term memory requires transcription and translation of new proteins, which are needed to grow new synaptic connections [116,117]. Long-term memory formation requires at least two waves of transcription after learning. This suggests that NR4A2 FT may contribute to the second of these waves of gene expression [118]. Thus, the expression of the IEG and NR4A2 TF plays a role in synaptic plasticity and memory formation by influencing the expression or regulation of other genes or pathways, such as *BDNF* and NF-κB [72]. Therefore, it is important to note that NR4A2 is crucial for long-term memory consolidation. In contrast, the initial encoding and retention of short-term memories seem less affected by NR4A2 activity [96]. In this regard, the regulation of *NR4A2* gene expression in memory-related processes has been emphasized particularly during learning, formation and consolidation of memories in brain regions such as the hippocampus, amygdala, nucleus accumbens, and cortex (insular and perirhinal) [97,98,99]. Thus, the mechanisms that regulate *NR4A2* gene expression are crucial for memory processes, particularly in memory formation in the hippocampus [13].

*NR4A2* expression is regulated by several mechanisms, including: (i) regulation by the CREB signaling pathway, a key pathway involved in memory formation [72]; (ii) the epigenetic mechanism of histone modification, such as acetylation. In this mechanism, histone deacetylases (HDACs), particularly HDAC3, remove histone acetyl groups from histones (deacetylation) in the *NR4A2* promoter region, thereby restricting *NR4A2* gene expression (Figure 5(B2)) [100].

This epigenetic mechanism of *NR4A2* gene expression regulation has been demonstrated in various studies, which have shown that: (i) *NR4A2* expression is promoted by memory-related processes accompanied by reduced HDAC3 occupancy at IEG promoters [98,101]; (ii) inhibition of HDAC3 activity (either pharmacologically or genetically) restores or continues cognitive functions related to memory processes and increases *NR4A2* expression [96,102,119]; (iii) decreased or altered expression (siRNA, shRNA knockdown) or activity (antagonist) of NR4A2 does not allow cognitive restoration associated with HDAC3 inhibition [102]. Taken together, these data show that HDCA3 decreases *NR4A2* expression, affecting long-term memory processes. Therefore, HDAC3 is a potent negative regulator of memory [120].

In particular, *NR4A2* regulation by the HDAC3 deacetylase domain has a selective role in specific brain regions that underlie long-term memory formation. This regulation influences different types of memory processes [98,121]. Thus, researchers have identified that HDCA3 negativity regulates *NR4A2* expression that impacts hippocampus-dependent long-term memories, including aversive [98], incidental [102] and reward [101,119] memories. These processes occur in brain regions such as the hippocampus, amygdala and nucleus accumbens, which are involved in learning, memory formation, consolidation and related behaviors.

Epigenetic regulation of *NR4A2* has been implicated in the mechanisms underlying age-related cognitive decline, and further research is needed to fully understand its role in this process. Kapris et al. (2019) [100] showed that aged rats exhibit greater variability in their cognitive abilities compared to young rats. Two groups were identified within the aged rats: those that were cognitively preserved (CP) and cognitive impaired (CI). CP rats performed similarly to young rats, while CI rats perform significantly worse. The *NR4A2* gene was induced during learning in young and CP rats, but not in CI rats. The study shows that HDAC3 limits *NR4A2* expression in the hippocampus of older rats, contributing to memory impairment. But removing HDAC3 restored *NR4A2* expression and improved memory in aged rodents. Additionally, overexpressing *NR4A2* in the hippocampus improves memory in hippocampal-dependent tasks, but not in hippocampal-independent ones. These findings suggest that memory deficits in cognitively impaired aged rats are associated with changes in genetic and epigenetic regulation of *NR4A2*. These results indicate that the epigenetic regulation of *NR4A2* by HDAC3 plays a significant role in age-related cognitive decline [100]. These findings suggest that increasing the expression or activity of the NR4A2 through therapeutic strategies may be effective in improving memory in the elderly [96].

#### 5.3.3. Epigenetic Regulation of *NR4A2* Gene Expression by Non-Coding RNA

Non-coding RNAs (ncRNAs) are functional RNA molecules that do not code for proteins but play a crucial role in regulating gene expression. They can be divided into infrastructural ncRNAs and regulatory ncRNAs. Constitutively expressed infrastructural ncRNAs include ribosomal, transfer, and small nuclear RNAs. Regulatory ncRNAs influence gene expression through a variety of mechanisms, including transcriptional, epigenetic and post-transcriptional regulatory processes. Among the regulatory ncRNAs are microRNAs (miRNAs), Piwi-interacting RNAs (piRNAs), small interfering RNAs (siRNAs) and long non-coding RNAs (lncRNAs) [122].

The ncRNAs can mediate the interaction between the epigenetic machinery and DNA by sequestering chromatin modifiers, guiding them to specific genomic locations or serving as molecular scaffolds to coordinate the binding of epigenetic complexes [123].

In particular, lncRNA are non-coding RNA molecules that are more than 200 nucleotides long. They are characterized by unique and specific expression patterns among tissues, as well as by the fact that they do not conserve sequence between species. LncRNAs regulate gene expression depending on their subcellular location (nucleus or cytoplasm) and are categorized according to their genomic origin and orientation. Enhancer-associated lncRNAs (elncRNA) are transcribed from enhancer regions of DNA [124].

Researchers have discovered that lncRNAs play a significant role in regulating gene expression at the epigenetic level. LncRNAs can interact with HDACs directly or indirectly through other proteins, forming complexes that influence gene expression. LncRNAs can influence the acetylation state of histones by recruiting HATs or HDACs to specific locations in the genome, thereby modulating gene transcription [124].

In particular, the LncRNA called ADRAM (activity-dependent lncRNA associated with memory) regulates the expression of *NR4A2* IEG in the context of neuronal activity and fear extinction learning [103]. Wei et al. (2022) [103] identified experience-induced *ADRAM* lncRNA that acts as a scaffold and a combinatorial guide. This lncRNA recruits the brain-enriched chaperone protein 14-3-3 to the promoter of the memory-associated *NR4A2* IEG, being a required process for fear extinction memory. *ADRAM* has been identified as an enhancer-derived RNA (eRNA). This lncRNA is upregulated in response to fear extinction learning and regulates *NR4A2* expression by directly interacting with its promoter. *ADRAM* guides 14-3-3 to the *NR4A2* promoter, which results in the time-dependent removal of HDAC3 and HDAC4. This is followed by the coordinated deposition of CBP (Figure 5(B3)) [103]. This increases *NR4A2* gene expression. The researchers emphasize that ADRAM is also expressed in other brain regions, including the hippocampus, cerebellum, and somatosensory cortex. This suggests that the regulation of *NR4A2* expression in other memory processes may be influenced by this eRNA [103].

### 5.4. Post-Transcriptional Mechanisms in NRA42 Gene Expression Regulation

Post-transcriptional mechanisms are crucial for regulating gene expression after the pre-RNA transcription but before mRNA translation into protein. These mechanisms fine-tune gene expression by affecting various aspects of mRNA processing, stability, localization, and translation efficiency.

#### 5.4.1. Alternative Splicing of *NR4A2* Pre-RNA and Its Regulation by CRTC

Alternative splicing (AS) is a process in which a single gene can produce multiple mRNA transcripts, leading to the creation of different protein isoforms. In this process, a pre-mRNA is processed in more than one way This results in the removal of different introns or the inclusion of different combinations of exons, and the consequent production of mature mRNAs with different sequences [125]. This mechanism allows for a single gene to encode for a variety of proteins, each of which may have a different function. AS can also affect the stability and other features of the mRNA. The majority of human genes undergo AS, and defects in AS are associated with disease [125,126].

On the other hand, different alternative splicing variants of the *NR4A2* mRNA have been identified in different species, such as humans, rodents and pigs [46,47,48,105,127]. Furthermore, at least 15 transcript variants of the *NR4A2* gene have been identified. Of these, 11 encode a protein and 4 encode nonsense mediated decay; however, their biological actions remain unclear [50]. These isoforms are generated by alternative splicing of exons 3, 5 and 7 [47,105,127]. In neurons, these isoforms have been observed to account for 20–35% of *NR4A2* transcripts. The resultant proteins show reduced transcriptional activity of the NR4A2 TF [127]. Most of the isoforms produced by the alternative splicing of *NR4A2* RNA can lead to the production of mRNA with different 3′UTRs lengths.

Alternative splicing is an important post-transcriptional mechanism that regulates *NR4A2* gene expression. This mechanism is influenced by CREB-regulated co-activators (CRTCs) [104].

In the brain, CRTCs act as sensors and integrators of hormonal, metabolic and neural signals, contributing to brain plasticity and communication between brain–body. This co-activator is both a potential genetic factor and a therapeutic target for psychiatric and neurodegenerative diseases [126,128]. CRTCs act as signal-dependent co-activators that regulate gene expression in response to cAMP. Under basal conditions, CRTCs are phosphorylated and are retained in the cytoplasm by 14-3-3 proteins. Increases in cAMP lead to the dephosphorylation of CRTCs, allowing them to translocate to the nucleus and bind to CREB. This enables the transcriptional regulation of CRE-containing genes [21].

In addition to acting as co-activators in transcriptional activation, Amelio et al. (2009) [104] showed that CRTCs selectively control pre-mRNA splicing alternative. This was identified by: (1) cAMP and calcium stimulation increased the process of alternative splicing of *NR4A2* in differentiated neuronal cells (ND-PC12); (2) the use of shRNA directed against CRTC reduced the selection of excision sites in these cells; (3) in hepatocytes, activation of *NR4A2* transcription does not result in significant changes to alternative splicing. Thus, researchers demonstrated that CRTC co-activators mediate alternative splicing of the *NR4A2* gene in a specific cellular context (Figure 5(C1)).

Furthermore, mutations in the functional domains of CRTCs demonstrate that they have distinct domains for transcriptional activation and AS [104]. These co-activators can induce AS without increasing transcriptional activation and vice versa. The alternative splicing of certain CREB target genes appears to be modulated by CRTCs via a conserved proline-rich domain. Despite lacking conserved RNA-binding domains, which are found in splicing factors, the CRTC splicing domain can act as a scaffold for assembling the protein complex involved in alternative splicing.

CRTCs are essential for regulating gene expression in response to environmental signals, demonstrating a dual regulatory function by acting in both transcriptional activation and pre-mRNA splicing site selection. However, these functions depend on the cell type or context of regulatory regions.

#### 5.4.2. *NR4A2* Gene Expression Through MicroRNAs

MicroRNAs (miRNAs) are non-coding RNA molecules comprising 18 to 25 nucleotides that play an important role in post-transcriptional gene expression [129]. In most cases, miRNAs interact with the 3′UTR of target mRNAs in order to induce mRNA degradation and translational repression (Figure 5(C2)) [130]. In this regard, several reports have described the transcriptional regulation of *NR4A2* by miRNA in DAergic neurons, cell death and cancer [105,106,107,108,109].

In embryonic pluripotent cells, miR-132 miRNA has been demonstrated to negatively regulate the differentiation of DAergic neurons by directly suppressing *NR4A2* expression. In this regard, the overexpression of miR-132 in a neurodegeneration model has been observed to result in a decrease in the expression levels of both *NR4A2* and brain-derived neurotrophic factor called BDNF in the midbrain [106,131]. Furthermore, increased miR-132 expression and diminished *NR4A2* mRNA levels were identified in peripheral blood cells of PD patients as compared to control group, as well as a negative correlation between these two RNAs [132].

On the other hand, studies have identified miR-145-5p miRNA as a regulator of *NR4A2* in microglia in an animal model of hypoxia and ischemia. In this animal model, a reduction in *NR4A2* expression resulted in neuronal death via TNF-α [19,133].

Moreover, different miRNAs have been identified that directly and negatively regulate *NR4A2* expression in cancer [108,109]. In particular, it has been demonstrated that miRNA-34c-5p directly and negatively regulates *NR4A2*. It is part of a possible feedback mechanism involving p53, miRNA-34 and *NR4A2* [108].

## 6. Dysregulation of the Expression of the *NR4A2* Gene in Animal Models and in Clinical Studies of Neurological and Psychiatric Disorders

The level of *NR4A2* gene expression is significantly associated with age-dependent neurodegeneration and cognitive function in animal models of PD, schizophrenia (SZ), AD, and attention deficit disorder [134,135,136,137]. Furthermore, clinical studies have identified *NR4A2* as a potential contributor to a range of dopamine neuron-related pathologies, including PD, cognitive deficits, developmental disorders, SZ, and AD [13,14,29,64,138]. In this manner, alterations in the expression of the *NR4A2* gene have been associated with various neurological and psychiatric diseases. However, altered *NR4A2* mRNA expression seems to occur in a disease-specific manner, suggesting that specific mechanisms are involved in regulating *NR4A2* expression and activity in each disease.

### 6.1. Parkinson′s Disease

Parkinson’s disease (PD) represents the second most prevalent neurodegenerative disease, following AD, and typically affects people over the age of 60. It is characterized by progressive loss of DAergic neurons in the substantia nigra, an important brain region of the nigrostriatal DAergic pathway [139]. The disease is characterized by both motor and non-motor symptoms [140]. A number of preclinical and clinical studies suggest that the *NR4A2* gene plays a significant role contributing to the pathogenesis of PD. In this context, several authors have suggested *NR4A2* regulation as a biomarker and potential PD therapeutic target.

In animal models, heterozygous *NR4A2* (*NURR1* (+/−)) mice showed a range of reductions in *NR4A2* gene and protein expression across different brain regions. In particular, a 39% decrease was observed in the substantia nigra pars compacta and the ventral tegmental area [141]. As a result, *NR4A2* (*NURR1* (+/−)) mice showed an increase in susceptibility to age-related dysfunction [142] and using MPTP neurotoxin [143], which is used as an animal model of PD.

A reduction in NR4A2 expression was observed in post-mortem samples of patients with PD, specifically in the substantia nigra pars compacta [144], in individuals with α-synuclein aggregates [145]. In this regard, a reduction in the level of expression of the *NR4A2* gene has also been observed in peripheral blood samples from patients with PD, which has been reported in several populations [132,146,147,148,149,150]. Furthermore, this decreased expression was accompanied by elevated levels of inflammatory cytokines and miRNA that regulate the *NR4A2* gene in PD patients [132,149].

The mechanisms that have been identified for the deregulation of *NR4A2* gene expression include α-synuclein, which negatively regulates *NR4A2* expression via NF-κB [75], as well as DNA methylation [113], and miRNAs [132].

With regard to DNA methylation, Kochmanski et al. (2022) [151] performed a genome-wide analysis of DNA methylation in enriched neurons obtained from the post-mortem parietal cortex of patients with PD [151]. The parietal cortex was used because this brain region shows pathological alterations in the late stages of PD. This study identified hypermethylation in an exon just downstream of an exon-intron boundary within the body of the *NR4A2* gene in male PD patients, but not in female PD patients. This hypermethylation of *NR4A2* gene in men with PD suggests that *NR4A2* gene dysregulation plays a role in the pathogenesis of PD and suggests the possibility for sex-specific differences in the development of this disease. Gender-related differences in *NR4A2* gene DNA methylation have also been observed in laboratory animals administered with insecticide dieldrin, a substance associated with an increased risk of PD. The study also identified specific differential methylation of the *NR4A2* gene in females [113] after exposure to the environmental toxin dieldrin, suggesting a mechanism that links environmental factors to altered *NR4A2* gene expression. Taken together, these studies suggest that sex-specific epigenetic regulation of the *NR4A2* gene may play a role in the risk of idiopathic PD.

On the other hand, altered *NR4A2* gene expression has been proposed as a potential therapeutic target for PD. In this context, it has been shown that NR4A2 agonist can restore the transcriptional activity of NR4A2 as a transcription factor, as well as the expression levels of this gene in an animal model of PD [152]. In this sense, neuroprotective treatments for DAergic neurons, such as EGb761 (a standardized extract of *Ginkgo biloba* leaves), have demonstrated that restoring *NR4A2* expression levels could be part of a neuroprotective mechanism [153].

Although the functional role of *NR4A2* is well known, it is not surprising that it is also involved in functions outside those of dopamine. Indeed, recent findings indicate that *NR4A2* exerts significant functions in numerous brain regions, encompassing neuroprotection and cognition [54,97,134].

### 6.2. Alzheimer′s Disease

Alzheimer’s disease (AD) is a neurodegenerative disorder that is the primary cause of dementia, which most individuals develop after the age of 65. It is characterized by a significant decline in cognitive abilities that results in an increased need for assistance with daily activities [154]. Glutamatergic neuronal activity is compromised in AD due to synapse destruction and neuronal death, and this deficit can affect memory, cognition and behavior, including cortical and hippocampal processing. Glutamatergic neurons, which use glutamate as their primary neurotransmitter, are particularly vulnerable in the hippocampus due to their critical role in excitatory neurotransmission and synaptic plasticity [155]. On the other hand, the pathological accumulation of glutamate can induce neurotoxicity.

The relationship between NR4A2 and AD has been investigated in animal models of this disease. For example, in a mouse model of AD known as 5X-FAD, *NR4A2* is highly co-expressed with amyloid β (Aβ) in the early stages, particularly in the subiculum and the frontal cortex [156]. In control and 5X-FAD mice, *NR4A2* expression is high in neurons but not in glia. Specifically, *NR4A2* expression in glutamatergic Aβ-positive hippocampal neurons degenerates in an age-dependent manner in 5X-FAD mice [134]. It has also been shown that *NR4A2* expression is significantly reduced when Aβ is administered to neuronal cells [157]. In addition, pharmacological activation of NR4A2 reduced Aβ plaque deposition in the subiculum and significantly ameliorated AD-like pathology in 5X-FAD mice, including neuronal loss, microglial activation and impaired neurogenesis in the adult hippocampus [134]. Taken together, these data suggest that a reduction in *NR4A2*-expressing glutamatergic neurons in the hippocampal formation may be associated with AD and that NR4A2 activation may be a promising therapeutic strategy for the treatment of AD [134].

In post-mortem samples from patients diagnosed with AD, a reduction in *NR4A2* expression was observed in the hippocampus and in individuals with Aβ deposition [158]. In comparison to control individuals without AD, patients with AD also exhibited a downregulation of the *NR4A2* gene in blood samples [146].

The precise mechanisms underlying altered *NR4A2* expression in AD remain unclear. However, in animal models of cognition, particularly memory and learning, histone deacetylases have been shown to be part of the mechanism involved in changes in the expression levels of this gene [100]. Similarly, the administration of NR4A2 agonists has been shown to improve memory and learning in animal models [100,159].

### 6.3. Neurodevelopmental Disorders

Neurodevelopmental disorders (NDDs) are a group of health conditions characterized by abnormal development of CNS, leading to brain malfunction later in life. Such health conditions may also present neuropsychiatric problems or impairments in motor skills, learning, language or nonverbal communication [160]. NDDs impose a significant burden on individuals, their families, and society in general due to their chronic and severe nature [161]. NDDs comprise intellectual disability (ID), communication disorders, autism spectrum disorder (ASD), attention-deficit hyperactivity disorder (ADHD), neurodevelopmental motor disorders (including tic disorders), and specific learning disorders.

A meta-analysis by Du et al. (2023) [162] identified significant alterations in neurometabolites such as N-acetyl-aspartate (NAA), gamma-aminobutyric acid (GABA), and glutamate + glutamine (Glx), in individuals with autism spectrum disorder. These alterations suggest dysfunctions in key processes such as neurotransmission, synaptic plasticity, and neuronal metabolism [162]. While the study itself does not address NR4A2 directly, previous research has implicated it with the regulation of genes involved in synaptic plasticity and the expression of glutamatergic receptors. In particular, NR4A2 has been proposed to play a role in glutamatergic synaptic plasticity [72], which may influence synaptic mechanisms altered in the autism spectrum disorder. In this context, several studies conducted in animal models and in patients have identified the *NR4A2* gene as a factor associated with NDD. For example, the *NR4A2*-deficient male mouse was associated with ADHD-like phenotype which may serve as a reliable tool for studying the behavioral phenotypes associated with ADHD and evaluating the clinical efficacy of potential therapeutic agents [135]. Furthermore, a prenatal stress model has implicated *NR4A2* expression as a mediator of the long-term effects of early life events. These changes, such as the dysregulation of *NR4A2* expression or the maintenance of *NR4A2* expression, may be associated with disease phenotypes [163] or resilience [164], respectively.

It should be noted that a number of genetic variants of NR4A2 have been linked to NDDs. Gabaldon-Albero et al. (2024) [64] conducted a recent systematic review of the literature [64] describing 26 patients with such variants who exhibit a heterogeneous neurodevelopmental phenotype. The phenotypic spectrum includes intellectual disability and/or developmental delay as the main features, as well as particular language impairment, dystonia or other movement disorders, and a high predisposition to neuropsychiatric disorders and/or epilepsy. These patients were also found to exhibit genetic heterogeneity in *NR4A2* variants ranging from single nucleotide variants (SNVs) to structural variants, such as copy number variants (CNVs) [65]. Among the SNVs, missense, frameshift, nonsense, and splicing variants have been identified. There do not appear to be any differences in the clinical presentation between structural variants CNVs and SNVs.

Studies using whole exome sequencing and functional validation have allowed us to understand the effects of *NR4A2* genetic variants on NDDs. Research has revealed that certain variants can lead to reduced gene expression or the production of a truncated protein. Basically, all of these variants lead to something called *NR4A2* haploinsufficiency [165], which is basically an alteration in its gene expression. Therefore, these variants are called pathogenic or likely pathogenic *NR4A2* variants. According to these data, the *NR4A2* gene could be considered a candidate gene for patients with suspected NDDs [165].

### 6.4. Schizophrenia

Schizophrenia (SZ) is a severe and chronic mental disorder, typically diagnosed in late adolescence. It led to significant disability and distress. According to the World Health Organization (WHO), it has a lifetime prevalence of ∼1%, and is considered one of the top ten causes of disability in developed countries. SZ is characterized by disturbances in thought, perception, and behavior that include hallucinations, delusions, paranoia, depression and cognitive impairments, among others [166]. Cognitive deficits are a core feature of schizophrenia, significantly contributing to the functional impairment associated with the disorder and remaining largely resistant to current treatments [167]. The development of these impairments, much like schizophrenia itself, arises from a complex interplay between genetic and environmental factors. It has been proposed that the expression of key genes must be linked to cognitive function in individuals with schizophrenia [168]. Alterations in gene expression can disrupt normal brain development and function, leading to dysregulation of neuronal signaling and synaptic dysfunction. These molecular changes may be associated with different neurotransmitters and neural circuits that are linked to cognitive deficits observed in schizophrenia [167,169], many of which are related to *NR4A2* gene expression.

In particular, *NR4A2* heterozygous (+/−), also known as *Nurr1* (+/−), mice have been proposed as an animal model to study some of the behavioral and molecular mechanisms underlying SZ [136,137]. These mice exhibit abnormal dopamine neurotransmission in the striatum and prefrontal cortex [137]. This DAergic imbalance-associated behavior has been linked to impaired emotional memory, increased spontaneous locomotor activity, heightened sensitivity to stress, and depression-like behavior [136,137]. Furthermore, male *NR4A2* (+/−) mice have been reported to have deficits in sensory gating using prepulse inhibition of the acoustic startle reflex [136], which is a common feature found in SZ patients [170].

In schizophrenia (SZ) patients, *NR4A2* expression in the dorsolateral prefrontal cortex (DLPFC) was found to be reduced in postmortem samples [171]. In addition, RNA-seq data from postmortem DLPFC tissue of SZ patients and healthy controls found that *NR4A2* expression levels were significantly reduced in patients [138]. This reduction may contribute to understanding the mechanisms involved in SZ pathophysiology. It is important to emphasize that DLPFC is a key part of the brain that controls executive functions, such as attention, behavior, and impulse inhibition. In SZ patients, the DLPFC is less activated during tasks that assess working memory and cognitive control.

Furthermore, genetic variants in the *NR4A2* gene have been found to be associated with the cognitive alterations present in the SZ [69,172]. Thus, genetic risk variants in the promoter, enhancer and intronic regions may be among the mechanisms involved in the dysregulation of *NR4A2* gene expression observed in SZ. Genetic variants found in a linkage disequilibrium (LD) block, together with variants in the promoter, enhancer and intronic regions within the *NR4A2* gene, have been associated with sustained attention in patients with SZ [69]. In addition, reduced *NR4A2* gene expression was correlated with impaired working memory in patients homozygous for a genetic variant located in the promoter region [172]. Based on these findings, the *NR4A2* gene has been proposed as a potential biomarker for cognitive endophenotypes in SZ.

### 6.5. Major Depressive Disorder

Major depressive disorder (MDD) is a chronic illness that affects an individual’s emotional, physical, and cognitive health [173]. MDD was the third leading cause of disease burden worldwide in 2018, and the WHO projects that it will become the leading cause by 2030 [174]. It is characterized by persistent depressed mood, anhedonia or loss of interest or pleasure in previously enjoyable activities, recurrent thoughts of death, feelings of guilt or worthlessness, lack of energy, poor concentration, changes in appetite, psychomotor slowing or restlessness, sleep problems, and other physical and cognitive symptoms [175]. Dysfunction of serotonin (5-HT) and dopamine signaling has been implicated in the etiology of MDD [176]. The dorsal raphe nucleus (DR) is a major source of 5-HT in the brain, and its dysfunction in 5-HT-related gene expression plays a significant role in the development of MDD [177]. It is a multifactorial and probably polygenic disorder. In this context, a significant decrease in *NR4A2* mRNA in DR has been reported in MDD patients [178]. Furthermore, genetic variants of *NR4A2* have been identified with susceptibility to MDD in the Caucasian population [68].

In particular, there are several preclinical models for studying depression in which the role of *NR4A2*, also known as *Nurr1*, has been described. One of the most widely used models is the forced swim test, a well-known and established preclinical animal model of depression-like behavior induced by an acute stressful event, since stress is an important factor in inducing depression. In this regard, it has been reported that *NR4A2* (*Nurr1*) heterozygous (+/−) mice exhibited a depression-like behavior, indicating difficulties in coping with stress [137]. Furthermore, it has been reported that using this particular model of depression-like behavior in adult mice led to a rapid increase in *NR4A2* (*Nurr1*) mRNA expression in specific brain regions following a second exposure to the forced swim test [179]. This response could be indicative of a compensatory mechanism that is developed to cope with depression-like behavior.

The findings of research studies have shown that animals responded differently to the forced swim test, which were classified as either vulnerable or resilient. After stress exposure, there was a significant increase in NR4A2 (Nurr1) protein levels in the hippocampus of vulnerable animals compared to the resilient group [180]. This differential response, which is associated with the behavioral phenotypes, may be attributed to the molecular response to stress, with NR4A2 (Nurr1) acting as a mediator of stress coping mechanisms. The molecular mechanisms underlying these two behavioral phenotypes are currently under study.

### 6.6. Substance Use Disorders

Substance use disorders (SUDs) are chronic, relapsing conditions in which substance use causes potential harm to physical or mental health, disability, and functional impairment for those affected and around them [181]. These conditions represent an important health, economic and social burden, being among the most significant causes of premature morbidity and mortality [182]. According to the Diagnostic and Statistical Manual of Mental Disorders (DSM-5-TR), SUDs are diagnosed using 11 criteria that fall into four basic categories: impaired control, physical dependence, social problems and high-risk use [183]. The causes, course, comorbidities, treatment use, and retention of SUDs differ between women and men, for a variety of reasons. These include biological, genetic, environmental and behavioral factors [184]. Worldwide, alcohol use disorders are the most common of all substance use disorders, followed by cannabis, opioid, and cocaine dependence [185]. A better understanding of the genetic mechanisms involved in the neurobiology of SUDs provides opportunities for the development of effective treatments and prevention strategies.

Cocaine addiction induces long-term neuroplastic changes [186] that, in turn, contribute to maladaptive behaviors, which are associated with transcriptional reprogramming of brain reward circuits [187].

In this regard, chronic cocaine administration has been shown to down-regulate the *NR4A2* gene in the rat ventral midbrain [188]. These results are consistent with the findings in postmortem human cocaine abusers, where the *NR4A2* gene is also reduced in the ventral tegmental area [189,190]. This decrease in *NR4A2* expression can be considered a regulatory response to the excess of dopamine neurotransmission caused by repeated cocaine exposure, which persists in the midbrain for at least two weeks after cocaine withdrawal [188]. This finding contributes to our understanding of the long-lasting brain changes associated with chronic drug use increased neural plasticity associated with prolonged exposure to drugs of abuse [191].

Prefrontal cortex (PFC)-dependent functions, such as executive function, explicit learning, and memory, are negatively affected in individuals with cocaine abuse as well as in experimental animal models of cocaine treatment [192,193]. Thus, *NR4A2* has been shown to exhibit dynamic expression changes in the PFC after repeated cocaine exposure in mice. An initial increase in *NR4A2* expression was observed, followed by a subsequent decrease after the last cocaine exposure. These transient changes after cocaine withdrawal may have played a role in the different stages of cocaine addiction [194]. The medial prefrontal cortex (mPFC) has been identified as a key region involved in the phenomenon of relapse after cocaine withdrawal. However, the underlying cellular mechanisms involved in these processes remain to be fully elucidated [195].

An important region of integration of the reward system, the nucleus accumbens (NAc), is the major component of the ventral striatum. Repeated cocaine exposure has been shown to induce coordinated changes in gene expression, which in turn drive plasticity in the NAc and promoting the development of addiction-like maladaptive behavior. Several studies have identified the role of transcription factors as critical mediators of cocaine-induced plasticity [196]. Most studies of epigenetic mechanisms involved in cocaine drug-taking and drug-seeking behaviors have focused on histone post-translational modifications in the NAc [197]. In this regard, it has been reported that memory formation is highly dependent on the regulation of the *NR4A2* gene by HDAC3 [100]. HDCA3 is one of four members of the human class I HDCAs. It regulates gene expression by deacetylating of histones and non-histone proteins [198]. Normally, HDAC3 binds to the promoters of *NR4A2*, repressing its expression. This regulatory function of HDAC3 may classify *NR4A2* as an important epigenetic effector gene [100]. In the NAc, *NR4A2* expression is increased in HDAC3 flox/flox (HDAC3 selectively depleted in the NAc) mice that are exposed to cocaine [101]. In wild-type mice, acquisition of a cocaine-induced place preference leads to a reduction in HDAC3 occupancy at the *NR4A2* promoter [101]. This phenomenon is associated with the increase in *NRA42* expression, thereby a link between *NR4A2* and cocaine-related behaviors is suggested. This heightened expression of *NR4A2* is involved in HDAC3-mediated cocaine context memory formation in mice [101].

The medial habenula (MHb), is one of several regions associated with depression, addiction, and nicotine withdrawal. This brain region may provide further insight into the mechanisms underlying relapse to cocaine use [199]. Studies have demonstrated that the expression levels of HDAC3 and *NR4A2* were highly expressed in this brain region [200,201]. Moreover, cocaine exposure in mice has been shown to elicit epigenetic regulation of the *NR4A2* gene during the reinstatement of cocaine-associated behaviors [201]. This suggests that HDAC3 is uncoupled from the *NR4A2* promoter in MHb as response to cocaine-induced reinstatement, which would imply an increase in *NR4A2* expression levels. These results suggest that HDAC3 acts as a molecular brake on memory by negatively regulating *NR4A2* [201]. It is also noteworthy that the expression of the dominant negative splice variant of *NR4A2*, termed *NURR2C*, in MHb cholinergic neurons provides a model to assess the effects of reduced *NR4A2* function in animal models [200,201]. Accordingly, Nurr2c expression in MHb has been shown to block cocaine-seeking reinstatement in mice. This finding facilitated the identification of the role of *NR4A2* in MHb in cocaine-induced associative memory processes. Furthermore, potential NR4A2 target genes associated with alterations in transcriptional networks related to addiction, neuroplasticity, GABAergic, and glutamatergic signaling have been identified by characterizing the molecular cascade resulting from reduced NR4A2 function [200]. These results further support the role of epigenetic regulation of *NR4A2* in MHb and its involvement in cocaine relapse.

## 7. Therapeutic Potential of Modulating *NR4A2* Gene Expression in Neurodegenerative Diseases and Psychiatric Disorders

NR4A2 is a promising neuroprotective and anti-inflammatory target in neurodegenerative and psychiatric diseases. However, because the effects of *NR4A2* dysregulation vary by brain region and disease, therapeutic strategies must be specific to each disease.

Among the most extensively studied approaches in preclinical research is the use of NR4A2 activators, which act at the NR4A2 TF or protein level. Examples of activators include prostaglandins (endogenous ligands), antimalarial drugs such as chloroquine and amodiaquine (synthetic ligands), and bicyclic compounds such as SA0025. Most studies on NR4A2 activators in neurodegenerative and psychiatric diseases have focused on PD, AD and cognitive impairments [134,152,159,202]. Comprehensive reviews of these NR4A2 activators have been carried out by other authors [13,203]. Nevertheless, these activators do not specifically target the brain regions involved in each pathology, and potential adverse effects cannot be ruled out due to the pleiotropic effect of NR4A2.

The mechanisms that regulate *NR4A2* gene expression have significant therapeutic potential for the treatment of neurodegenerative and psychiatric diseases. As shown in Table 1, various preclinical and clinical studies of *NR4A2* expression provide evidence for supporting the identification of its regulatory mechanisms in these diseases. Although some mechanisms of *NR4A2* genetic dysregulation may be shared across diseases, the affected brain regions appear to be specific to each disease. Therefore, understanding the timing and tissue-specific patterns of *NR4A2* dysregulation is a key factor for elucidating the pathophysiology of each disease. It is also critical to consider the influence of other genetic and environmental factors contributing to these pathologies.

With the advent of new genome editing and genetic engineering technologies such as CRISPR/Cas, CRISPR/dCas, and gene therapy, a new window of opportunity has opened not only for investigating gene expression mechanisms but also for developing tools that enable specific treatments for neurodegenerative and psychiatric diseases [205,206,207]. These technologies offer the potential to act on specific tissues or even a specific region of the brain [208,209,210]. This represents a major advantage over conventional systemic pharmacological approaches.

In general, gene therapy, in its most elementary form involves introducing genetic material into target cells using either non-viral or viral vectors, with the aim of treating or preventing diseases by correcting or supplementing defective genes [207]. Adeno-associated viruses (AAV) are among the most widely used viral vectors. This is because they are safe, naturally efficient at delivering genes to cells, and can produce sustained transgene expression in multiple tissues [208]. On the other hand, the CRISPR-Cas system consists of clustered regularly interspersed short palindromic repeats (CRISPR) and CRISPR-associated proteins (Cas), was discovered to be a prokaryotic defense mechanism that leads to an adaptive immune response [205]. CRISPR/Cas is a gene editing system that enables precise DNA modification at a specific site within a living organism (deleting, inserting or correcting sequences). It uses a Cas protein, such as Cas9, as molecular scissors and a customizable guide RNA (gRNA) to locate and cleave the target DNA in a sequence-specific manner. This DNA can then be replaced or repaired [211].

In contrast, CRISPR/dCas-based systems provide a powerful approach to modulate gene expression and genome function without altering the DNA sequence. This technology uses a genetically modified Cas enzyme that has lost its cutting activity (dCas) but retains its ability to recognize specific DNA via RNA guidance (gRNA) [206]. The dCas can bind to functional domains of other proteins important in epigenetic mechanisms, including those involved in the acetylation, deacetylation or methylation of histones, as well as DNA methylation. Thus, this system can perform epigenetic regulation of a specific gene [207].

In particular, the study of gene expression mechanisms and like potential therapeutic of *NR4A2* has been carried out using CRISPR/Cas technology and VAA vectors, especially in the field of oncology, but also in PD, AD and substance use disorders [199,201,212,213,214]. However, this field offers endless possibilities for future research into the mechanisms that regulate *NR4A2* expression [201], especially epigenetic mechanisms involved in neurodegenerative and psychiatric diseases, along with potential therapeutic interventions targeting the regulatory pathways of the *NR4A2* gene.

## 8. Discussion

The NR4A2 TF plays an important role in regulating the expression of several genes involved in mechanisms related to proper brain function, including neurodevelopment, protection and homeostasis of different neuronal cell populations, with the highest levels reported in dopaminergic neurons [8,9,19]. In particular, the regulation of gene expression by NR4A2 TF occurs through genomic and non-genomic pathways. However, the mechanisms that regulate its transcriptional activity are diverse, with the regulation of *NR4A2* gene expression being one of the most important.

The gene that encodes this TF, *NR4A2*, also known as *NURR1*, exhibits homology across different species [46,47]. This gene consists of a promoter region, eight exons and seven introns [48]. Depending on the cellular context, this gene can be stably regulated or induced as an IEG, leading to pleiotropic physiological effects. Furthermore, its expression is tightly regulated by extracellular signals. Consequently, the mechanisms that regulate *NR4A2* gene expression occur at multiple levels, through transcriptional, epigenetic and post-transcriptional mechanisms. Among the epigenetic mechanisms that regulate *NR4A2* expression are DNA methylation, histone deacetylases (HDACs) and non-coding RNA (ncRNA) (Figure 5) [100,105,113].

Several animal models and clinical studies have linked *NR4A2* gene dysregulation to neurodegenerative diseases and psychiatric disorders. Thus, *NR4A2* gene dysregulation has been implicated in PD, AD progression, SZ, substance abuse (alcohol and cocaine), NDDs and cognitive impairment [7,13,14,15,16,17,18]. However, these changes in *NR4A2* expression occur in a disease-specific, cell type-associated manner and can be associated with the disease stage, suggesting the existence of disease-specific mechanisms underlying *NR4A2* dysregulation.

As discussed in this review, it is important to note that the mechanisms associated with *NR4A2* regulation are still being explored. In particular, some neurodegenerative diseases and psychiatric disorders have been studied more than others. Thus, we can highlight mechanisms regulating *NR4A2* expression in some CNS diseases.

This review has the following limitations: first, because of the limited number of studies conducted in humans, the review predominantly relies on studies conducted in animal models, which could restrict the generalization of the findings to human biological systems. It is also important to remember that the studies performed in animal models of the different diseases are the basis for the hypothesis that *NR4A2* is involved in different human diseases, but as we know, this does not mean that animal models reflect the reality of human diseases. Likewise, excluding literature not written in Spanish or English may have limited the representativeness of the available evidence. The narrative approach introduces a risk of bias in the selection and synthesis of studies. Finally, the scarcity of recent data from single-cell RNA sequencing techniques related to NR4A2 could limit our understanding of its functional specificity according to cell type. These limitations underscore the need for future research with broader coverage to strengthen the validity and applicability of the findings.

## 9. Conclusions

This revision encourages us to continue to further explore the diverse mechanisms that regulate *NR4A2* expression in different diseases and disorders, including those associated with neurodevelopmental, neurodegenerative, addiction, and mental disorders. Understanding these mechanisms will allow us to identify therapeutic targets for neurological diseases and psychiatric disorders.

The accumulated evidence supports the translational relevance of *NR4A2* as a potential biomarker and a therapeutic target. However, significant gaps remain in our understanding of its specific mechanisms of action, epigenetic regulation, and function in different cell types.

In this context, the combination of genetic and epigenetic large-scale data with bioinformatic tools represents a significant breakthrough in identifying genetic regulatory mechanisms. One example is TRAPT (Transcription Regulator Activity Prediction Tool) tool, which uses epigenomic data to predict transcriptional regulators (TRs) in specific contexts, such as neurodegenerative diseases [215]. This tool could contribute not only to characterizing the regulatory activity of NR4A2 TF, but also to inferring the TRs involved in *NR4A2* gene expression. These TRs, such as transcription cofactors and chromatin regulators, may mediate regulatory signals between the promoters and distal enhancers of the *NR4A2* gene, thereby regulating its expression, particularly in the context of neurodegenerative and psychiatric diseases.

On the other hand, it is a priority to advance toward specific clinical applications. Thus, it is necessary to conduct preclinical studies with NR4A2 agonists, evaluating their efficacy and safety in animal models of the aforementioned diseases and disorders. Likewise, longitudinal studies in humans, especially in relevant clinical cohorts, are required to validate the potential of *NR4A2* as a diagnostic and or prognostic biomarker.

At the same time, the impact of epigenetic modifications on the expression and function of NR4A2 should be investigated, considering its possible reversibility as a therapeutic strategy. It is also key to explore cell-type-specific delivery technologies that allow NR4A2-based therapies to be directed toward the involved neuronal or glial populations, reducing off-target effects in uninvolved tissues.

These lines of research could open the way for personalized treatment of neurodegenerative diseases and psychiatric disorders, reinforcing the role of NR4A2 in the field of translational medicine.

Therefore, we can conclude that *NR4A2* is a potential therapeutic target for neurological and/or psychiatric diseases, due to its key role in the survival of dopaminergic neurons, as well as in the regulation of neuroinflammation and neuronal plasticity, among other functions. In this context, it is important to highlight that recent advances in network pharmacology are providing a deeper understanding of drug mechanisms of action, especially in complex diseases such as neurological and psychiatric diseases.

Currently, the development of new drugs requires an interdisciplinary approach that integrates biology, pharmacology and bioinformatics, as proposed by network pharmacology. This discipline allows us to explore how drug affect multiple genes, proteins, and signaling pathways simultaneously in complex diseases. This contrasts with traditional pharmacology, which focuses on a single molecular target. This type of study has been performed, for example, for the treatment of intracerebral hemorrhage [216]. This represents a valuable contribution to the search for drugs in complex neurological and psychiatric diseases associated with NR4A2 dysfunction.

## Figures and Tables

**Table 1:** Comparison of NR4A2 gene expression dysregulation in neurodegenerative and psychiatric disorders.

Neurodegenerative Disease/ Psychiatric Disorder	*NR4A2* Expression in Patients	Relevant Studies in Animal Models	Potential Mechanisms Implicated in Gene Regulation
Parkinson’s disease (PD)	In patients with PD: Postmortem brain samples showed decreased in *NR4A2* mRNA expression in regions such as the substantia nigra, which plays a key role in movement, and in individuals with α-synuclein aggregates [144,145]. Reduced *NR4A2* mRNA levels in peripheral blood were accompanied with elevated levels of inflammatory cytokines and miR-132 [132,149]. Neurons from the postmortem parietal cortex exhibited sex-specific DNA methylation changes in the *NR4A2* gene [151].	*NR4A2* heterozygous mice are more susceptible to the neurotoxin MPTP, which is a model of PD [143]. An NR4A2 agonist restores both the transcriptional activity of NR4A2 as a transcription factor and its gene expression levels in a PD animal model [152]. In mice administered insecticide dieldrin, a compound associated with an increased risk of PD, sex-specific DNA differential methylation of the *NR4A2* gene was observed [113].	The presence of synuclein, which negatively regulates *NR4A2* expression via NF-κB, therefore decreases *NR4A2* mRNA expression [29,89]. The microRNA miR-132 directly targets and represses *NR4A2* gene expression [106,131]. The impact of alterations on DNA methylation is not yet fully understood. It is unclear how these alterations impact *NR4A2* expression or if they play a part in disease progression. No research has been conducted on this subject.
Alzheimer’s disease (AD)	In patients with AD: Postmortem samples revealed reduced *NR4A2* expression in the hippocampus and in individuals with Aβ deposition [158]. Peripheral blood samples from AD patients also showed downregulation of the *NR4A2* gene [146].	*NR4A2* expression is significantly reduced when Aβ is administered to neuronal cells [157]. In 5X-FAD mouse model of AD, *NR4A2* is highly co-expressed with amyloid β (Aβ), particularly in the subiculum and the frontal cortex [134]. Pharmacological activation of NR4A2 reduced Aβ plaque deposition in the subiculum and significantly ameliorated AD-like pathology in 5X-FAD mice [134].	The precise mechanisms underlying altered *NR4A2* expression in AD remain unclear. However, in animal models of cognition, particularly memory and learning, histone deacetylases have been shown to be part of the mechanism involved in changes in the *NR4A2* gene expression levels [100].
Neurodevelopmental Disorders (NDD)	In patients with NDD, evidence suggests the presence of pathogenic or likely pathogenic *NR4A2* variants. These variants lead to reduced gene expression or the production of a truncated NR4A2 protein [64].	*NR4A2*-deficient male mice were associated with ADHD-like [135].	*NR4A2* haploinsufficiency [64].
Schizophrenia (SZ)	In SZ patients: Reduced *NR4A2* expression was observed in the dorsolateral prefrontal cortex (DLPFC) of postmortem simples [138,171]. Variants in *NR4A2* gene have been associated with the cognitive alterations observed in the SZ [69,172]. Hypermethylation of the *NR4A2* gene was found in females with major psychosis, compared to female control subjects [204].	*NR4A2* heterozygous (+/−) mice, also known as Nurr1(+/−) have been proposed as an animal model for studying some of the behavioral and molecular mechanisms underlying SZ [136,137].	It is unclear how DNA methylation impacts *NR4A2* expression or if they play a part in disease progression. No research has been conducted on this subject. However, in animal models of cognition, particularly memory and learning, have shown that histone deacetylases are part of the mechanism involved in changes in the *NR4A2* gene expression levels [100].
Major depressive disorder (MDD)	In MDD patients: A significant reduction in *NR4A2* mRNA expression has been reported in the dorsal raphe nucleus [178]. Genetic variants of the *NR4A2* gene have been identified as risk factors for MDD in Caucasian populations [68].	*NR4A2* (NURR1) heterozygous (+/−) mice exhibited a depression-like behavior, indicating difficulties in coping with stress [137].	The impact of genetic variants on *NR4A2* expression is not fully understood. No research has been conducted on this subject.
Substance use disorders	Postmortem human individuals with history of cocaine and heroin abuse: The ventral tegmental area showed reduced *NR4A2* gene expression [189,190]. This decrease may be considered a regulatory response to excessive dopaminergic neurotransmission induced by repeated cocaine exposure.	In mice, heightened *NR4A2* expression is involved in the formation of cocaine context memory in the nucleus accumbens via HDAC3 [101]. Cocaine exposure in rats has been shown to elicit epigenetic regulation of the *NR4A2* gene by HDAC3 during the reinstatement of cocaine-associated behaviors [200,201].	The role of HDAC3 in the epigenetic regulation of *NR4A2* in medial habenula and nucleus accumbens [101,200,201].

## Data Availability

No new data were created or analyzed in this study. Data sharing is not applicable to this article.

## Abbreviations

The following abbreviations are used in this manuscript:

Aβ	amyloid β
AD	Alzheimer′s disease
ADHD	Attention-deficit hyperactivity disorder
AF1	Ligand-independent activation function domain 1
AF2	Ligand-dependent activation function domain 2
ASD	Autism spectrum disorder
BDNF	Brain-derived neurotrophic factor
CNS	Central nervous system
CP	Cognitively preserved
CI	Cognitive impaired
CNVs	Copy number variants
CREs	Cis-regulatory elements
CREB	cAMP response element-binding protein
CRTC	CREB-regulated transcription co-activators
CRTC1	CREB regulated transcription co-activator 1
CTCF	CCCTC-binding factor
DAergic	Dopaminergic neurons
DBD	DNA-binding domain
DMR	Differential methylation region
DR	Dorsal raphe nucleus
DSM-5-TR	Diagnostic and Statistical Manual of Mental Disorders
eRNA	Enhancer-derived RNA
GSK-3	Glycogen synthase kinase 3
HDAC	Histone deacetylases
HDAC 3	Histone deacetylases 3
IEG	Immediate early gene
ID	Intellectual disability
LBD	Ligand-binding site
LBD	Ligand-binding domain
LD	Linkage disequilibrium
lncRNAs	long non-coding RNAs
MDD	Major depressive disorder
mPFC	Medial prefrontal cortex
5mC	5-methylcytosine
miRNAs	microRNAs
miRNA-132	microRNA 132
miRNA-145-5p	microRNA-145-5p
miRNA-34	microRNA-34
mRNA	Messenger ribonucleic acid
MHb	Medial habenula
NAc	Nucleus accumbens
ncRNAs	Non-coding RNAs
NDDs	Neurodevelopmental disorders
NF-κB	Nuclear Factor Kappa B
NR4A2	Nuclear receptor subfamily 4 group A member 2
NR4A2 mRNA	messenger RNA transcript of NR4A2
PD	Parkinson’s disease
PFC	Prefrontal cortex
piRNAs	Piwi-interacting RNAs
5-HT	Serotonin
SZ	Schizophrenia
SNVs	Single nucleotide variants
SUDs	Substance use disorders
TF	Transcription factor
UTR	5′-untranslated region
WHO	World Health Organization
